# Thumbprint purpura from disseminated strongyloidiasis in a heart transplant recipient

**DOI:** 10.1016/j.jdcr.2025.10.044

**Published:** 2025-10-30

**Authors:** Marissa Yaldo, Camilla A. Cascardo, Tran H. Do, Severine Cao

**Affiliations:** a Wayne State University School of Medicine, Detroit, Michigan; b Department of Dermatology, Michigan Medicine, Ann Arbor, Michigan

**Keywords:** heart transplant, *Strongyloides*, subcutaneous ivermectin, thumbprint purpura

## Introduction

*Strongyloides stercoralis* is a gastrointestinal helminth found in soil that infects humans through larval penetration of the skin. The larvae travel to the lungs and ascend the tracheobronchial tree, where they are coughed up and swallowed to reach the small intestine.^1^ While immunocompetent individuals are typically asymptomatic and harbor low parasite burden, immunosuppressed patients, particularly solid organ transplant recipients, are at an increased risk for severe infection manifesting as hyperinfection syndrome or disseminated strongyloidiasis.^1^^,^^2^ We report a case of disseminated strongyloidiasis in a heart transplant recipient presenting with classic dermatologic findings who was successfully treated with compassionate use of subcutaneous ivermectin.

## Case report

A 62-year-old male with a history of cardiac transplant 2 months prior presented to the emergency department with progressive dyspnea and hypoxia. He was found to be in septic shock secondary to *Klebsiella pneumoniae* bacteremia and bacteriuria. Bronchoscopy revealed diffuse alveolar hemorrhage. Infectious workup to this point was unrevealing, including a negative respiratory panel, negative cultures from his bronchoalveolar lavage, and a negative stool ova & parasite (O&P). Given clinical deterioration and concern for infection not captured by conventional microbiologic testing, plasma microbial cell-free DNA (Karius) testing was pursued, which returned positive for *Strongyloides.* Of note, serologic testing revealed normal eosinophil counts.

**Fig 1:**
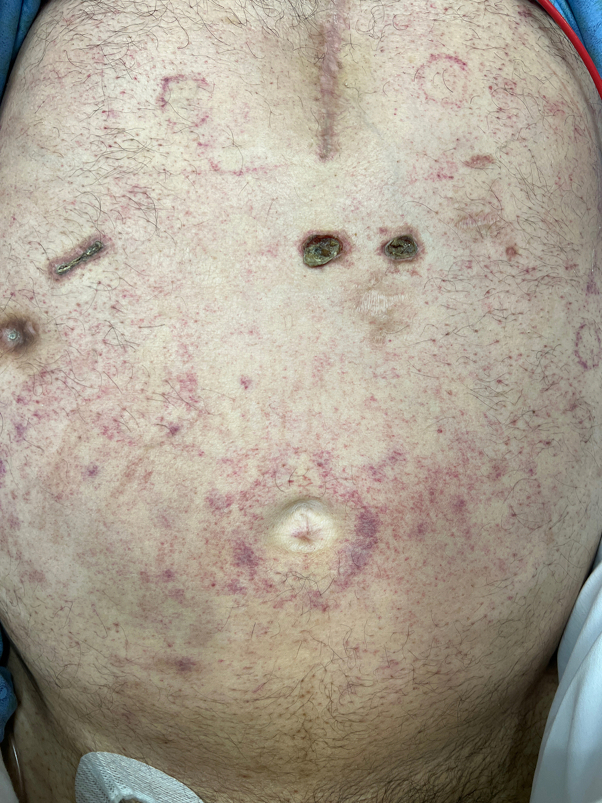
Faint purpuric macules scattered across the abdomen and in areas where adhesives were removed, consistent with “thumbprint purpura”.

**Fig 2:**
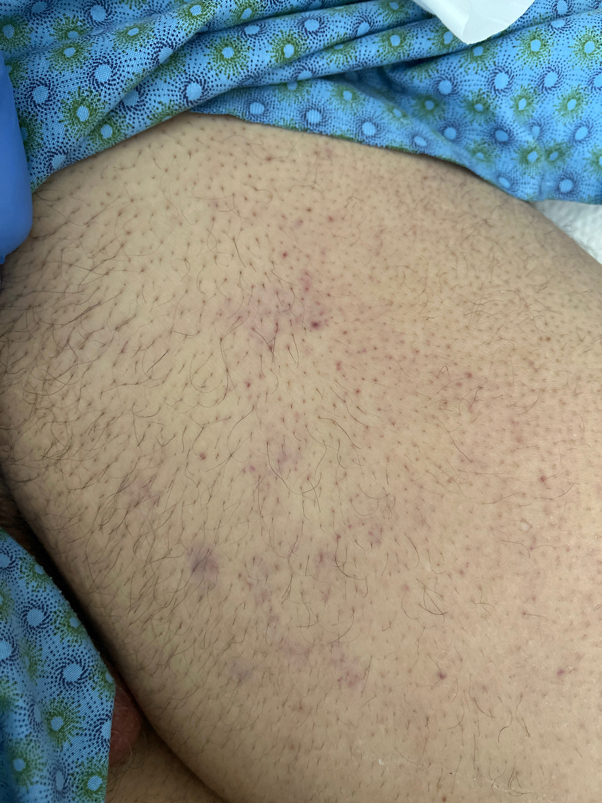
Faint purpuric macules of the left proximal thigh.

**Fig 3:**
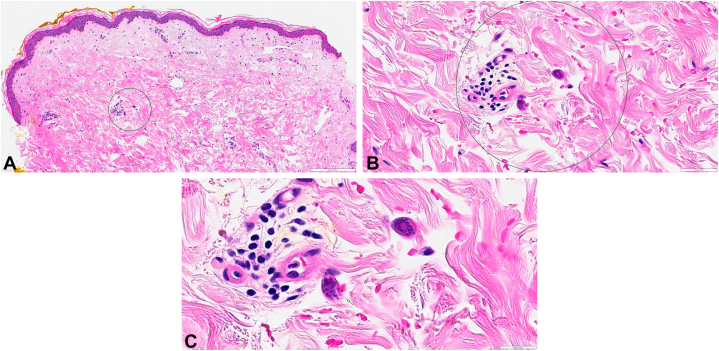
**A-C,** H&E stain at 10×, 40×, and 80× showing larvae and egg-like forms within the dermis, most suggestive of *Strongyloides*. *H&E*, Hematoxylin and eosin.

At this time, dermatology was consulted for evaluation of a rash on the abdomen and thighs. Physical examination revealed faint purpuric macules and patches located on the chest, periumbilically (Fig 1), and on the proximal thighs (Fig 2). On the chest, the purpura was noted to follow the outline of prior adhesives. Punch biopsy of the left upper thigh (Fig 3, *A*-*C*) revealed larvae and egg-like forms within the dermis, most suggestive of *Strongyloides*.

Rereview of his bronchoalveolar lavage revealed a rare focus morphologically compatible with *Strongyloides,* and repeat stool O&P returned positive, further confirming the diagnosis.

The patient was started on oral ivermectin 200 mcg/kg daily and oral albendazole 400 mg twice daily. Due to the life-threatening risk of infection and concern for impaired gastrointestinal absorption of oral therapy, the infectious disease team sought Food and Drug Administration (FDA) approval for compassionate use of subcutaneous ivermectin 200 mcg/kg. Stool O&P testing was closely followed and subcutaneous ivermectin was continued for 2 additional weeks following the first negative result. The patient’s immunosuppressive regimen was reduced. Piperacillin–tazobactam was also continued to reduce the risk of bacterial dissemination secondary to larval migration. The patient ultimately received 5 weeks of oral ivermectin, 3 weeks of oral albendazole, and 5 weeks of subcutaneous ivermectin. He successfully cleared the infection and achieved complete recovery while preserving graft function.

## Discussion

Disseminated strongyloidiasis is a rare condition that typically affects immunocompromised individuals, including transplant recipients.^1^^,^^2^ In immunocompromised patients, previously dormant *Strongyloides* parasites residing in the gastrointestinal tract can reactivate, even in individuals with exposure decades earlier.^2^ Once reactivated, the infection can disseminate from the gastrointestinal tract to other organs, including the skin and sites outside the autoinfection cycle.^1^ Along with the parasite, patients may experience translocation of gastrointestinal bacteria such as *Escherichia coli*, *Klebsiella pneumoniae*, and *Pseudomonas* species, leading to sepsis.^1^ This likely occurred in our patient, who presented with *Klebsiella pneumoniae* bacteremia.

Strongyloidiasis in the setting of immunosuppression may be suspected in patients who have resided in endemic areas, which include East Asia, sub-Saharan Africa, Latin America, and the Carribean.^3^ Cases of strongyloidiasis derived from organ donation have also been reported.^4^^,^^5^ In our patient, reactivation of a dormant infection was suspected given his prior residence in Palestine, although a donor-derived infection could not be excluded. Given the high mortality in transplant recipients, screening at-risk donors and recipients for *Strongyloides* antibodies may be beneficial to enable prophylactic treatment.

Disseminated strongyloidiasis can affect any organ but typically presents with fever, respiratory symptoms, and gastrointestinal distress.^6^ Dermatologic findings may serve as the most specific clinical indicator of disseminated disease and present with truncal petechiae and purpura that radiate centrifugally around the periumbilical area and on the upper thighs, often referred to as “thumbprint purpura”.^7^, ^8^, ^9^ This finding is believed to result from larval migration through vessel walls, causing hemorrhage.^8^^,^^9^ Correspondingly, biopsy of purpuric lesions demonstrates larvae.^6^^,^^7^ We noted the accentuation of purpura around sites of prior adhesives. While not previously described in the literature, we propose that this finding may be due to local pressure and trauma at these sites, facilitating the local disruption of dermal vasculature by migrating larvae.

The gold standard for diagnosis is detecting larvae in stool through microscopic examination.^2^ However, this has varying sensitivity and may not always detect active infection. For this reason, multiple stool samples may be needed. Larvae may also occasionally be identified in respiratory secretions, ascitic fluid, cerebrospinal fluid, or urine.^2^ Other methods, such as enzyme-linked immunosorbent assay for detecting *Strongyloides*-specific immunoglobulin G antibodies may be used but cannot distinguish between current and prior infection. Molecular testing includes next-generation sequencing which has been increasingly used for diagnosis.^2^^,^^10^ These tests may be of particular utility in immunosuppressed patients, who may have varying results with serum or stool testing due to a decreased immune response.^2^ Next-generation sequencing exhibits high sensitivity and specificity and identifies active *Strongyloides*, but use is limited due to availability and cost.^2^^,^^10^

While eosinophilia can be used as a diagnostic marker for parasitic infection, it may not occur in immunosuppressed patients or those taking steroids due to a suppressed eosinophilic response,^9^ as seen in our patient. Notably, those with absent eosinophilia tend to experience poorer outcomes.^9^

The mortality rate of disseminated *Strongyloides* has been reported to be as high as 85%.^9^ Treatment in transplant patients requires cautiously reducing immunosuppression while monitoring for graft rejection. Repeated doses of oral ivermectin should be administered, with ongoing stool testing to monitor for parasite clearance.^2^ In disseminated disease, gastrointestinal absorption may be limited due to paralytic ileus, small-bowel obstruction, or profuse vomiting and diarrhea.^2^ Subcutaneous ivermectin circumvents gastrointestinal absorption but is currently only FDA-approved for veterinary use.^2^ Thus, FDA approval for compassionate use is required.^2^ This combination successfully treated our patient, and we suspect that it played a key role in his survival.

## Conflicts of interest

None disclosed.
